# The association of brightness with number/duration in human newborns

**DOI:** 10.1371/journal.pone.0223192

**Published:** 2019-10-01

**Authors:** Cory D. Bonn, Maria-Eirini Netskou, Arlette Streri, Maria Dolores de Hevia

**Affiliations:** 1 Department of Psychology, University of British Columbia, Vancouver, BC, Canada; 2 Integrative Neuroscience and Cognition Center (CNRS UMR 8002), Université Paris Descartes, Paris, France; Cornell University, UNITED STATES

## Abstract

Human neonates spontaneously associate changes in magnitude across the dimensions of number, length, and duration. Do these particular associations generalize to other pairs of magnitudes in the same way at birth, or do they reflect an early predisposition to expect specific relations between spatial, temporal, and numerical representations? To begin to answer this question, we investigated how strongly newborns associated auditory sequences changing in number/duration with visual objects changing in levels of brightness. We tested forty-eight newborn infants in one of three, bimodal stimulus conditions in which auditory numbers/durations increased or decreased from a familiarization trial to the two test trials. Auditory numbers/durations were paired with visual objects in familiarization that remained the same on one test trial but changed in luminance/contrast or shape on the other. On average, results indicated that newborns looked longer when changes in brightness accompanied the number/duration change as compared to no change, a preference that was most consistent when the brightness change was congruent with the number/duration change. For incongruent changes, this preference depended on trial order. Critically, infants showed no preference for a shape change over no shape change, indicating that infants likely treated brightness differently than a generic feature. Though this performance pattern is somewhat similar to previously documented associations, these findings suggest that cross-magnitude associations among number, length, and duration may be more specialized at birth, rather than emerge gradually from postnatal experience or maturation.

## Introduction

Identifying and combining related environmental signals across sensory modalities is a central challenge of multisensory perception. How humans construct and tune internal models for organizing information from multiple sensory signals is thus a key question for perceptual and cognitive development. A handful of studies suggest that humans get a head start from basic integration capacities inherited from evolution. At birth, humans come equipped with a number of mechanisms for identifying related sensory signals across modalities, including tactile-to-visual transfer of shape and texture information [1, 2], the use of audiovisual synchrony to match faces with voices and tones [3] as well as arbitrary audiovisual pairings [4] (cf. [5]), matching of facial gestures with speech sounds [6], and audiovisual matching of numerical information [7]. These results suggest that newborns possess a number of amodal or abstract representations that facilitate crosstalk between domain-specific sensory inputs or perceptual dimensions.

Recently, it has been shown that newborns possess a special kind of multisensory mapping capacity: the ability to associate representations of relative quantity across length, duration, and number [8]. Specifically, when an auditorily presented numerical quantity and/or event duration increases, newborns prefer a simultaneously presented increase in visual length; conversely, when number and duration decrease, newborns prefer visual length to decrease. Some studies also show that associations among different quantitative dimensions may not be unique to humans [9, 10], suggesting that newborns’ associations among quantities may either have ancient evolutionary roots or that stable environmental pressures have led to its spontaneous emergence as a homoplasic trait. However, it is not yet clear whether newborns’ preference for matched quantity changes across length, duration, and number reflects a specialized mechanism linking those particular domains or whether these preferences at birth are more general. A specialized mechanism might indicate a perceptual system biased to detect links among percepts that are often closely related in the environment [11, 12]; for example, it takes longer to travel larger distances given a constant rate of motion. At the implementational level of analysis, this bias/specialization may be reflected in shared or overlapping neural or cognitive resources [13, 14] (cf. [15]). A more general-purpose mechanism—one that extends similar associations to other dimensions such as brightness, loudness, hue saturation, warmth, weight, etc.—might indicate that newborns are simply matching quantities using an abstract code that facilitates cross-talk. This code may be dimensionless (e.g., ratios and ranks [16]) or reflect a propensity to re-code non-spatial quantities as spatial representations (e.g., [10, 17, 18]. In this paper, we begin to explore how specialized the newborn quantity-association system is: is it unique to space, time, and number or does it extend to other quantities?

Evidence from experiments conducted on older infants suggests that some quantitative representations may be more easily associated together than others–and that this heterogeneity persists into adulthood. For example, Srinivasan and Carey [19] found that 9-month-old infants more easily associate positively correlated lengths and durations, rather than negatively correlated ones, and that these positive (but not negative) length-duration relationships boost adults’ recognition memory for specific stimulus pairs. In addition, infants can generalize or match coarse-grained, relative-magnitude descriptions akin to “more” vs. “less” and “increasing” vs. “decreasing” across quantities of length, duration, and number (9-month-olds [20] and 8-month-olds [21]). On the other hand, 9-month-old infants do not associate any pairings of length and loudness [19] and mapping between number and brightness is far less robust in 8-month-old infants, who show no baseline preference for number and brightness to change in the same direction, while at the same age infants show such a preference for number-length pairings. In addition, while infants generalize order from number to length, they fail to do so from number to brightness [21, 22]. Some evidence indicates that this less robust relationship with brightness persists later in childhood [23] and adulthood [24, 25] (cf. [26]).

Given that relations among spatial, temporal, and numerical representations appear to be privileged beginning in infancy with respect to signed associations with other quantitative dimensions (i.e., loudness, brightness), our specific research question is whether this special status is acquired in infancy as a result of post-natal experience and/or maturational processes, or whether this special status is present from birth. In fact, privileged associations among these quantities in infancy may result from experience with natural correlations present in the environment: relationships among number, space and time may be more common and/or informative than those across other dimensions. On the other hand, it might be possible that privileged relationships between some dimensions reflect intrinsic core properties of newborns’ perceptual or cognitive capacities that make these quantities more readily connected to one other, and therefore this special status might be present from birth, before any significant experience with the environment.

To distinguish between these two possibilities, we extended the methods of de Hevia et al. [8], where newborns were shown to create expectations of congruency between changes across number and/or duration on the one hand and spatial extent on the other. In the present study, we therefore tested whether newborns’ preference for number (with redundant duration information) and length to change in the same direction (both increasing or both decreasing) generalizes to the pairing between the dimensions of number/duration and brightness. Brightness was a natural choice because there is evidence that it is less readily associated with number in older populations (older infants, children, and adults), and because it is easy to manipulate experimentally, whereas other dimensions like loudness do not fit well within the bimodal stimulation paradigm used in these studies with newborns. In this design, newborns experience an auditorily presented number (confounded with duration) paired with a shape of a specified brightness in a familiarization trial. In a subsequent set of 2 test trials, infants experience a new number/duration. On one of those test trials, no accompanying visual change occurs. On the other trial, one of 3 possible changes occurs: (1) brightness changes in parallel to number/duration, (2) brightness changes in the opposite direction of the number/duration change, or (3) the shape of the object changes.

If newborns’ number/duration-to-brightness mapping capacity operates in the same way as their number-length and number-duration mapping capacity, based on previously published results, we expect newborns to prefer trials with a visual change over no visual change only when brightness changes match the sign of the number/duration changes. However, newborns should show no such preference when number/duration and brightness change in the opposite direction. This would suggest that sign-matched changes across number, length, and duration are not privileged among quantities.

If newborns show no difference in preference across the two conditions but simply prefer to attend to stimuli with any arbitrary visual change accompanying a numerical/durational change (i.e., more changes are more interesting to attend to), then they should show equivalent preference patterns across all three conditions. This would unambiguously indicate a privileged status for sign-matched changes across number, length, and duration, to the exclusion of any type of association with other dimensions.

Alternatively, newborns may simply expect that brightness levels change regardless of sign at the same time as changes in number/duration, but still group quantities together as a category (see also [27] for a related hypothesis that change relative to background is what matters for brightness and loudness). In this case, associations among length, time, and number would be special for generating the expectation of sign-matched changes at birth, whereas a larger class of quantities may simply require quantity change.

## Experiment

**Fig 1:**
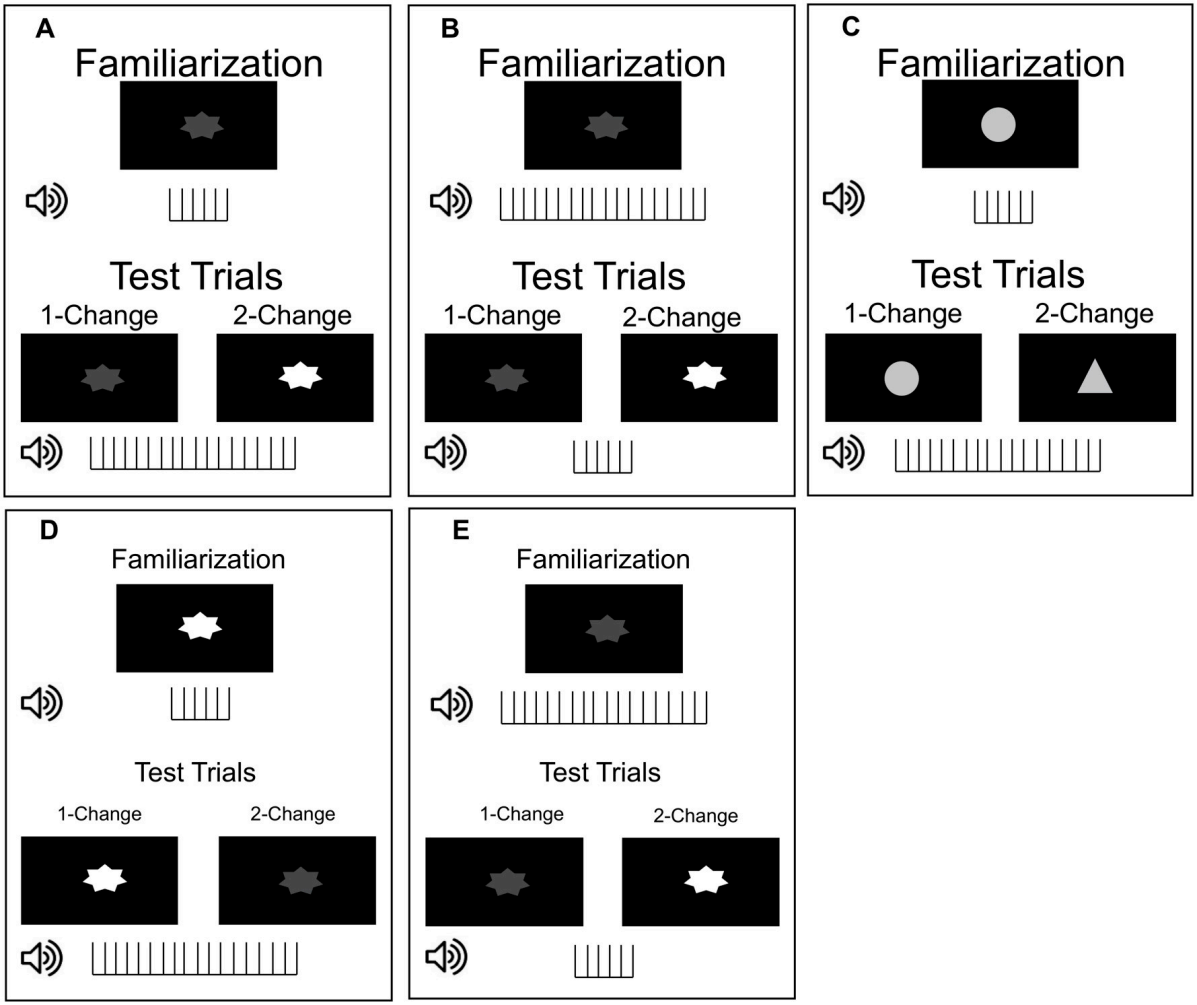
Example stimuli. Vertical tick marks below images indicate the occurrence of a single syllable (total of 6 or 18 syllables). A. Congruent Condition/Increasing. B. Incongruent Condition/Increasing. C. Shape-Change Control. D. Congruent Condition/Decreasing. E. Incongruent Condition/Decreasing.

We examined newborns’ responses to simultaneous changes in number/duration and either luminance/visual brightness or object shape. Infants were assigned to one of three familiarization conditions: (1) the Congruent condition, in which the infants were familiarized to either a low-luminance stimulus paired with a small number of syllables (and thus a short-duration stimulus overall) or a high-luminance stimulus paired with a large number of syllables (and thus a long-duration stimulus overall); (2) the Incongruent condition, in which infants were familiarized to either a low-luminance stimulus paired with a large number of syllables (long duration) or a high-luminance stimulus paired with a small number of syllables (short duration); and to control for visual novelty, (3) a Shape-Change control condition, in which the shape of a circle rather than a star with varying luminance values was paired with a small or large number of syllables (short or long durations, respectively). See Fig 1 for examples of stimuli.

Following familiarization, infants experienced two test trials with a novel numerical/durational stimulus; infants familiarized to a small number of syllables (short duration) experienced a large number (long duration) in the two test trials, while infants familiarized to a large number of syllables (long duration) were tested with a small number (short duration). The crucial manipulation was in the type of visual change: during 1-Change test trials, no visual feature change accompanied the numerical/durational change; during 2-Change trials, both the visual feature (brightness or shape) changed as well as the number of syllables (duration) heard.

If newborns prefer brightness to increase or decrease in parallel with increases or decreases in number/duration, subjects in the Congruent condition should look longer during the test trial with both auditory and visual changes than during the test trial with only an auditory change. In contrast, subjects in the Incongruent condition should show no difference in looking time across test trials, as they are not given the opportunity to experience congruent changes (i.e., both dimensions either increasing or decreasing) from familiarization to test. This outcome would support the hypothesis that number, length, and duration mappings are not privileged at birth.

If subjects prefer brightness to change simultaneously with number/duration but without a preference for a change in the same magnitude direction, then subjects in the Congruent *and* Incongruent conditions should look longer during test trials with changes in both sensory modalities. In the case where evidence is consistent with the second prediction, another explanation is possible: newborns could simply prefer trials with a greater amount of stimulus change. If that were the case, then newborns should also prefer test trials in the Shape-Change condition in which the stimulus changes in both sensory modalities. This would constitute evidence consistent with the second hypothesis that sign-matched changes for number, length, and duration are privileged at birth, to the exclusion of other representations.

Finally, subjects may prefer brightness to change with number/duration regardless of sign, yet show no preference for shape to change in parallel with number/duration. This would constitute evidence consistent with the third hypothesis that number, length, and duration are privileged with respect to the expectation of sign-matched mappings, but that less restricted mappings are expected for other quantities.

## Materials and methods

The study protocol was approved by the ethics committee at Université Paris Descartes. We obtained written, informed consent from parents prior to testing each newborn.

### Participants

A total of 48, healthy, full-term newborn infants successfully completed the study (16 for each condition; 21 female; mean age = 50 hours/21 minutes; *SD* = 19 hours/45 minutes; range = 12 hours/24 minutes to 89 hours/44 minutes). Twenty-eight other newborns were recruited and tested but excluded for sleepiness (11), lack of interest in the stimuli (5), crying (6), looking times exceeding the maximum trial length on both test trials (4), experimenter error (3), and mother interference (1).

All infants had an Apgar score of 10 after 5 minutes. Infants were recruited directly inside the maternity ward, with the authorization of the director of the maternity department at L’Hôpital Bichat-Claude Bernard, Paris, France. All infants were tested in the presence of the mother.

The sample size for each Condition was set in advance to match de Hevia et al. [8]. Data from Experiment 1 reported in that study indicate that power to detect an interaction between Condition (Congruent vs. Incongruent) and Trial Type (1-Change vs. 2-Change) at the same effect size to be at .95 for a sample size of 16 at *α* = 0.05.

### Stimuli

Numerosities with duration information were presented auditorily in the form of sequences of syllables, repeated either 6 or 18 times [for studies using the same syllable sequences, see [7, 8, 28]. Eight different syllables pronounced by male and female speakers were used. The duration of individual syllables was similar in both auditory sequences so that the total duration of the sequences was shorter for the 6-syllable sequence (1.4 *s*) and three times longer for the 18-syllable sequence (4.2 *s*). Therefore, the auditory sequences contained information from both numerosity and temporal information. The silence between two sequences varied randomly between 2 and 3 *s*.

In the Congruent and Incongruent conditions, visual stimuli consisted of a star (8*cm* × 8*cm*; 7.6° × 7.6°) moving stroboscopically around the center of the screen at intervals of 1 s. For the lower brightness level, the star had a luminance of 6 *cd*/*m*^2^ and for the higher brightness level the star had a luminance of 46 *cd*/*m*^2^, a change well above known luminance thresholds for newborns [29]. Both stars were presented against a black background. The brightest display therefore had both the highest luminance and the greatest brightness contrast, since the latter has been found to determine the psychological direction of the continuum: higher contrasts are associated with larger numbers [26] and longer lines [21, 23].

In the Shape-Change Control condition, the visual stimuli consisted of a circle in familiarization and a gray triangle at test (with a constant luminance of 31 *cd*/*m*^2^) presented against a black background; newborns are known to be able to discriminate these shapes [30].

### Design and procedure

The paradigm had a familiarization phase (60 *s*) immediately followed by a test phase. During familiarization, each infant heard sequences of one numerosity/duration paired with one visual stimulus. Test trials contained either one change (auditory only) or two changes (auditory and visual). The order of the test trials was counterbalanced across participants and familiarization types. Each test trial continued until the baby looked away for two, consecutive seconds or until a maximum trial length of 60 *s*.

In the Congruent condition, infants were familiarized to sequences of 6 syllables and a low-luminance star or familiarized to sequences of 18 syllables and a high-luminance star. In the Incongruent condition, infants were familiarized to sequences of 6 syllables and a high-luminance star or familiarized to sequences of 18 syllables and a low-luminance star. In the Shape-Change Control condition, infants were familiarized to sequences of 6 or 18 syllables paired with a gray circle.

During the two test trials in each of the Brightness manipulations (Congruent and Incongruent conditions), the auditory numerosity/duration changed (from 6 to 18 or vice versa). The test numerosity/duration was paired with either the same brightness level presented in familiarization (1-Change trial) or a novel brightness level (2-Change trial; 6 to 46 *cd*/*m*^2^ or vice versa). During the test trials in the Shape-Change Control condition, the auditory numerosity/duration change was accompanied by no visual change (1-Change trial) or a triangle (2-Change trial).

Infants were placed in an infant seat, 60 cm from a 22-inch monitor, and an experimenter stood behind the infant to monitor for potential signs of discomfort. A second experimenter situated behind the monitor coded the newborn’s looking times online (via a separate monitor with a live feed from a camera pointed at the infants’ faces) by pushing a button on the keyboard when the baby looked at the screen.

### Coding

Another experimenter coded looking times offline, from the video recording played at slow speed. Because newborns’ looks are not always easy to code (if the eyes are not wide open), and online coding was necessarily permissive (for example, if an infant sneezed, the experimenter did not stop the trial), a third experimenter coded looking times offline when the two first coders’ judgments differed by more than 5 *s* (34% of all trials). All coders were blind to the visual, but not the auditory stimuli. The analyses reported are based on the average of the two closest measurements for each trial (the correlation between the two measurements was *R*^2^ = 0.98 for all conditions). This means that the final measurement could be an average of the online coder and one offline coder, if no third coder was necessary, or, if a third coder was used, it could be either of the two possible pairs of coders, one online or both offline. If the infant presented signs of distress or drowsiness, the experimenter who coded the looking times online terminated the study before it was completed. Offline coders, who were blind to the experimental conditions, decided when an infant who had completed the study was too drowsy/fussy to be included in the data analyses.

## Results

### Raw-data visualization and assumption checks

**Fig 2:**
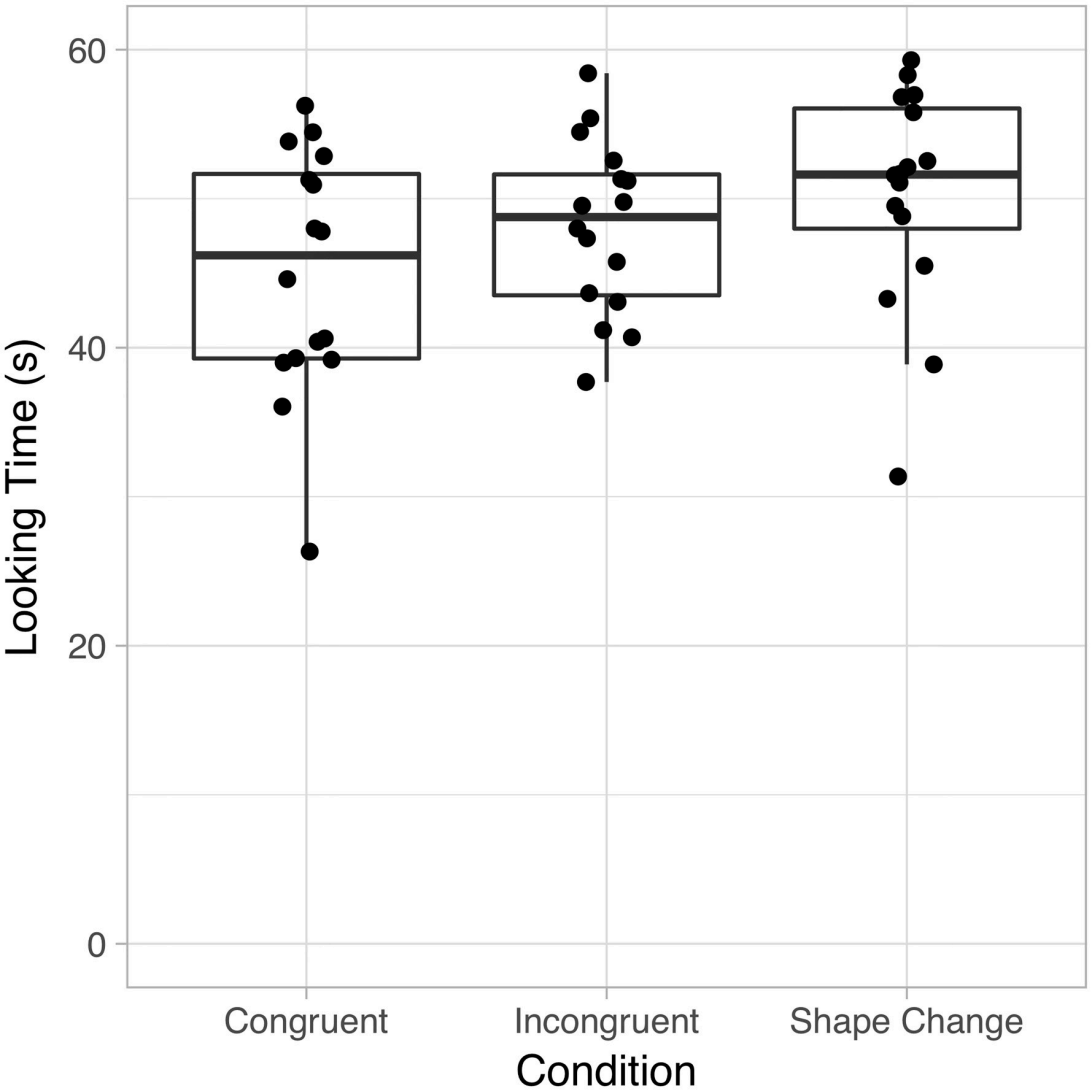
Looking times during familiarization. Boxes represent the interquartile range (IQR) of the data; the median is indicated by the middle bar. The whiskers represent the highest or lowest values up to 1.5 times the IQR from the hinges.

**Fig 3:**
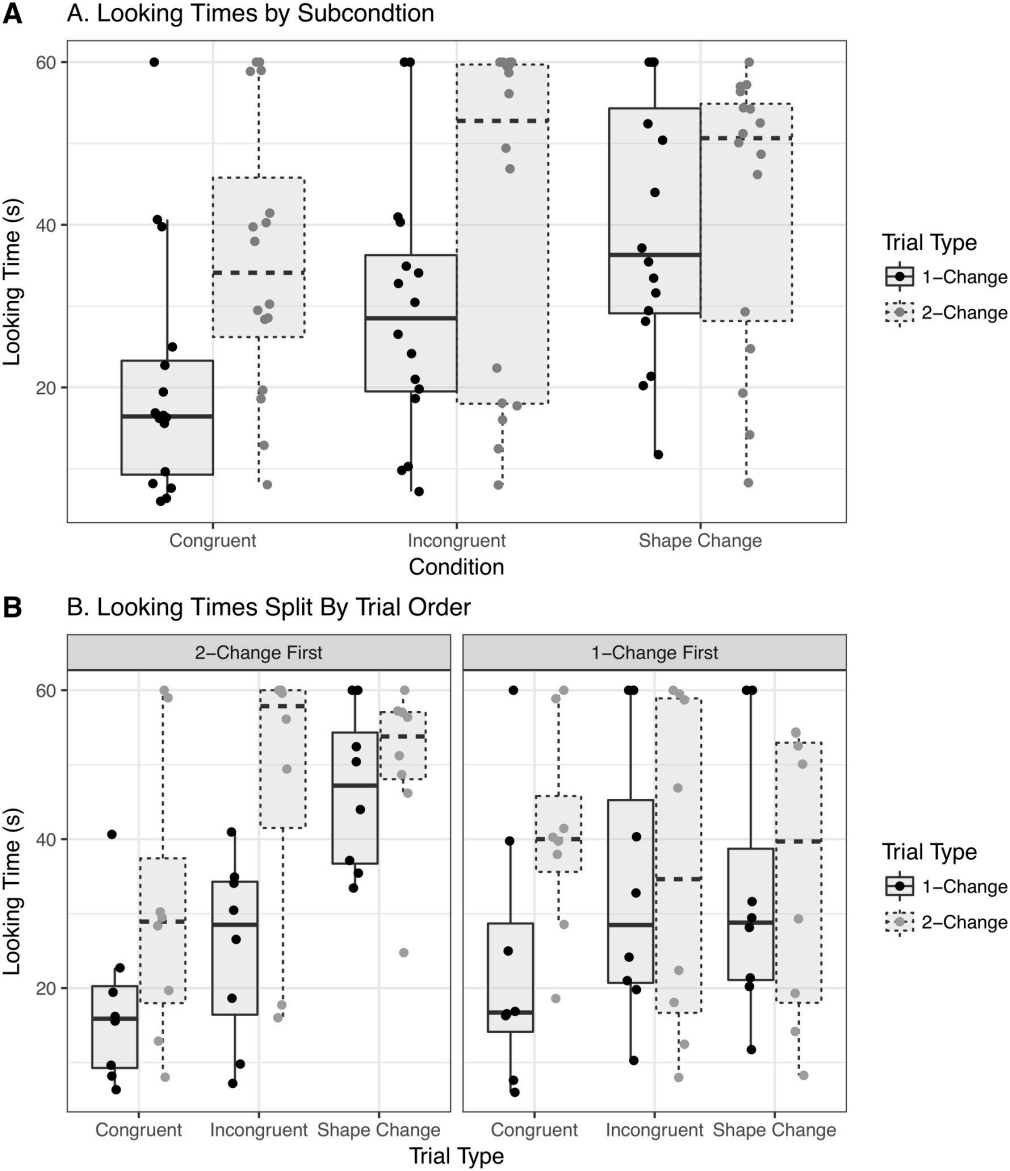
Test-trial looking times. Raw data indicated by individual dots. Boxplots give the median, IQR, and whiskers represent up to 1.5 times the IQR from the hinges. A. Collapsing across orders, differences between the 1-Change and 2-Change trials are similar for Congruent and Incongruent conditions. Looking times in the Shape-Change condition are similar across Trial Type. B. When divided according to trial order, newborns in the Incongruent condition look equally long at the 1-Change and 2-Change trials when 1-Change trials appear first. Newborns in the Shape-Change condition look for shorter durations overall when the 1-Change trial occurs first.

Raw looking times during Familiarization for each condition are shown in Fig 2 and looking times during test trials are shown in Fig 3. We quantitatively assess these patterns with a set of statistical analyses that include the originally planned analyses of variance on raw looking times as well as more complex models to more accurately assess the level of uncertainty in effect sizes under varying assumptions.

Prior to conducting the main statistical analyses to quantify these patterns, we performed a number of assumption checks which revealed deviations from the assumptions necessary for valid inferences from the classical ANOVA model, including a right-skewed distribution of looking times and a relatively large number of observations censored at 60 *s*. The term ‘censored’ is used here to mean that the event of interest, looking termination, would have taken place after the period of observation is over. See Appendix A in S1 Appendix for details on the assumption checks.

### Statistical models

Most studies of infant looking times, including analyses from previously published work in our lab on newborns as well as on older infants, report repeated-measures ANOVA with post-hoc *t*-tests to assess the contribution of each experimentally manipulated factor to the variance in the data and to give estimates of pairwise comparisons of looking-time differences across conditions. We report this analysis here first. We then build similar models within a Bayesian framework containing further refinements of the assumed likelihood function to more accurately assess the amount of uncertainty associated with estimates of group differences.

As a first step, we verified that Familiarization did not significantly vary as a function of Condition; an ANOVA failed to reveal a significant effect of Condition on familiarization looking times (*F*(2, 45) = 2.05, $\eta_{p}^{2}=0.08$, *p* = 0.14). However, subjects varied widely in their level of attentiveness within all familiarization conditions, indicating the potential need to include this variable as a predictor in the final statistical models to account for individual variability in interest in the test stimuli.

**Fig 4:**
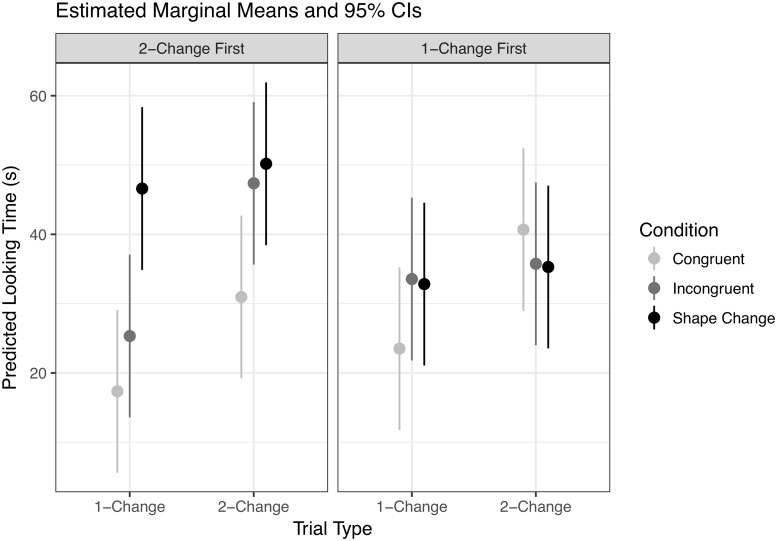
Estimated means and 95% confidence intervals for the classical, within-subject ANOVA.

A repeated-measures ANOVA with raw test-trial looking times as the dependent measure and Condition, Trial Type, and First Test Trial as factors confirmed that the newborns responded to the experimental manipulations: (1) there was a significant main effect of Condition (*F*(2, 42) = 4.33, $\eta_{p}^{2}=0.17$, *p* = 0.02) and (2) a main effect of Trial Type (*F*(1, 42) = 10.4, $\eta_{p}^{2}=0.2$, *p* < 0.005), but (3) no significant main effect of First Test Trial (*F*(1, 42) = 0.55, $\eta_{p}^{2}=0.01$, *p* = 0.46). The only potentially important interaction in this ANOVA is the marginal two-way interaction between First Test Trial and Condition (*F*(2, 42) = 3.14, $\eta_{p}^{2}=0.13$, *p* = 0.05). Fig 4 shows the estimated marginal means derived from the model.

An analysis of the Tukey-corrected simple effects associated with the marginal interaction conducted with the emmeans package [31] showed that for newborns who experienced 1-Change trials first, there were no significant differences in looking time across levels of Condition, averaging across Trial Type (*p* values above 0.14). For newborns who experienced 2-Change trials first, the only difference in Condition levels was between newborns assigned to the Congruent Condition and newborns assigned to the Shape Change Condition: on average, newborns in the Shape Change Condition looked 24 *s* longer than those in the Congruent Condition (*M* = −24.24, *SE* = 6.31, *t*(42) = −3.84, *p* < 0.005). Note that degrees of freedom reflect model-based pairwise comparisons with pooled variances rather than *post-hoc t*-tests on subsets of data; see online documentation of emmeans.

Counterbalanced orders were intended to disentangle potential order effects from Condition effects. Here the marginal interaction complicates the interpretation of these results. It is possible that an order effect diluted an overall Trial Type by Condition interaction that would indicate qualitatively different expectations across conditions, so in an exploratory analysis we calculated the simple effect of Trial Type at different levels of Condition, first averaging over trial order and then broken down by test-trial order. However, because the two-way Trial Type by Condition interaction and the three-way Trial Type by Condition by First Test interaction were not significant, differences across condition and subcondition should be interpreted with caution: though a simple effect may be significant in one level but not another, this does not necessarily mean that the two simple effects are significantly different from one another under the assumption of normally distributed residuals.

Newborns looked longer in the 2-Change trials than in 1-Change trials in the Congruent condition (*M* = −15.39, *SE* = 5.47, *t*(42) = −2.81, *p* < 0.01) and in the Incongruent condition (*M* = −12.12, *SE* = 5.47, *t*(42) = −2.22, *p* = 0.03), but not in the Shape-Change condition (*M* = −3.02, *SE* = 5.47, *t*(42) = −0.55, *p* = 0.58).

When broken down by trial order, newborns in the Congruent condition looked marginally longer in 2-Change trials compared to 1-Change trials in the 2-Change first subcondition (*M* = −13.61, *SE* = 7.73, *t*(42) = −1.76, *p* = 0.09) and in the 1-Change First condition (*M* = −17.16, *SE* = 7.73, *t*(42) = −2.22, *p* = 0.03), indicating potentially consistent behavior across trial orders. Newborns in the Incongruent condition looked significantly longer in 2-Change trials compared to 1-Change trials in the 2-Change First order (*M* = −22.04, *SE* = 7.73, *t*(42) = −2.85, *p* < 0.01), but not in the 1-Change First order (*M* = −2.21, *SE* = 7.73, *t*(42) = −0.29, *p* = 0.78). Finally, newborns in the Shape-Change condition did not apparently look longer in 2-Change trials compared to 1-Change trials in either order (2-Change First: *M* = −3.58, *SE* = 7.73, *t*(42) = −0.46, *p* = 0.65; 1-Change First: *M* = −2.47, *SE* = 7.73, *t*(42) = −0.32, *p* = 0.75). A further difference between 2-Change First and 1-Change First trials (*M* = 14.34, *SE* = 6.31, *t*(42) = 2.27, *p* = 0.03) in that Condition rules out the possibility that the failure to distinguish between 1-Change and 2-Change trials was driven by a failure to discriminate the shapes. In other words, newborns who got 2-Change trials on trial 1 looked longer than those who got 1-Change trials (i.e., no visual change), implying successful discrimination.

This exploratory analysis suggests that the order effect present in the Incongruent condition may be diluting the Trial Type by Condition interaction. If this were the case, then removing the data from the Incongruent condition from the analysis should unmask a Trial Type by Condition effect. An additional exploratory ANOVA without the data from the Incongruent Condition weakly confirms this hypothesis with a marginal Trial Type by Condition interaction (*F*(1, 28) = 3.76, $\eta_{p}^{2}=0.12$, *p* = 0.06).

### Interim discussion

From this analysis, we tentatively conclude that newborns consistently prefer 2-Change over 1-Change trials in the Congruent condition regardless of trial order, prefer 2-Change over 1-Change trials in the Incongruent condition when 2-Change trials are presented first, and consistently show no preference for 2-Change over 1-Change trials in the Shape-Change condition. This pattern of looking times is most consistent with the interpretation that newborns expect auditory number/duration to change in parallel with visual brightness but might also accommodate changes in brightness in the opposite direction, given a particular presentation order. Otherwise, Incongruent changes in brightness appear to be treated similarly to changes in shape.

### Assessing uncertainty under varying structural assumptions

Given that the pattern of looking times was more complex than expected, with apparent order-dependent effects in the Incongruent condition, we wished to examine whether the substantive conclusions change when (1) accounting properly for the previously noted violations of distributional assumptions and (2) accounting for the possibility that the division of Condition into 8-member subgroups based on trial order gives rise to spurious effects. That is, are improper assumptions and small samples masking a stronger relationship between number/duration and brightness at birth with spuriously large order-dependence in one condition? Or, conversely, is the relationship we observe possibly an artifact of a combination of small sample sizes with improper assumptions?

To flexibly modify varying assumptions and to better control the possibility of finding spurious effects in small sample sizes, we developed Bayesian versions of the model with weakly informative priors based on previously published experimental data to appropriately quantify the amount of uncertainty about group differences. Bayesian analysis offers control over the range of possible effect sizes with weakly informative priors and thus reduces overinterpretation of sampling error in a more principled way than *p*-value correction or model penalization. A common, alternative solution to handling assumption violations is non-parametric analyses; however, because they can only handle relatively simple inferences (one-way group comparisons) and have the downside of discarding information in the outcome measure, we opted not to use them here. Another solution designed to handle censoring without making assumptions about the shape of the likelihood is a survival analysis using a frailty term for subject-level effects (i.e., a random effect), but given that it is less readily interpretable for infant researchers due to its rarity in looking-time studies and given that this analysis leads to similar conclusions as the censored log-normal model below, we do not report this analysis in the main text 9for an example without frailty, see [32]; see S1 Supporting Information for this analysis).

For these analyses, we shifted to building regression models rather than implementing analyses of variance for both practical reasons and for ease of interpretation. Multilevel regression models with sets of categorical predictor variables can be thought of as a generalization of mixed-design ANOVA and classical regression techniques [33, 34]. They require *a priori* coding of group contrasts, which allows for immediate interpretation of the size of group differences upon model fitting and therefore reduce some of the need for extra calculation of comparisons not explicitly specified in the model. For details on model specification, see Appendices A2 and A3.

#### Model types

We examined 2 models estimated with maximum likelihood without accounting for censoring to provide a bridge between the original ANOVA and the Bayesian regression models. The first was a regression model on raw looking times wih the above predictors and the second was a regression model on log-transformed looking times. It is important to note that transforming the raw data does not correspond exactly to assuming the residual error term is log-normal, though for the purpose of bridging the frequentist and Bayesian analyses the assumption here is a reasonable simplification. The coefficients are given in Table 1 for reference and the associated summary analyses of variance are given in Table 2; the model of log-transformed data had a better (pseudo) *R*^2^ of 0.50, while the model on the raw looking times (equivalent to the classic mixed ANOVA) had a worse *R*^2^ of 0.38, calculated conditional on the random effects using the piecewiseSEM package [35]. Marginal *R*^2^ for fixed effects were similar (log-transformed: *R*^2^ = 0.29; raw: *R*^2^ = 0.29), showing that the benefit of log-transforming data may mostly improve the model fit to the distribution of individual subject means.

**Table 1:** Maximum likelihood models.

	Raw LT (s)					Log-Transformed LT				
*Coefficient*	*Estimate*	*SE*	*df*	*t*	*p*	*Estimate*	*SE*	*df*	*t*	*p*
(Intercept)	34.95	1.68	48	20.81	0.0000	3.37	0.06	48	52.55	0.0000
Familiarization Time	2.16	1.83	48	1.18	0.2432	0.07	0.07	48	1.05	0.3004
Congruent—Shape Change	-11.58	4.31	48	-2.69	0.0099	-0.44	0.16	48	-2.65	0.0109
Incongruent—Shape Change	-5.11	4.15	48	-1.23	0.2235	-0.21	0.16	48	-1.30	0.2007
2-Change − 1-Change	10.18	2.95	48	3.45	0.0012	0.33	0.10	48	3.49	0.0011
2-Change First − 1-Change First	3.25	3.39	48	0.96	0.3428	0.10	0.13	48	0.76	0.4534
(Cong—Shape) × (2-Change − 1-Change)	12.37	7.23	48	1.71	0.0938	0.61	0.23	48	2.59	0.0126
(Incong—Shape) × (2-Change − 1-Change)	9.10	7.23	48	1.26	0.2143	0.28	0.23	48	1.19	0.2405
(Cong—Shape) × (2-Change First − 1-Change First)	-19.93	8.46	48	-2.35	0.0227	-0.71	0.32	48	-2.21	0.0319
(Incong—Shape) × (2-Change First − 1-Change First)	-11.64	8.27	48	-1.41	0.1657	-0.39	0.32	48	-1.23	0.2242
(2-Change − 1-Change) × (2-Change First − 1-Change First)	5.80	5.91	48	0.98	0.3313	0.18	0.19	48	0.96	0.3426
(Cong—Shape) × (2-Change − 1-Change) × (2-Change First − 1-Change First)	-4.65	14.46	48	-0.32	0.7490	-0.25	0.47	48	-0.54	0.5929
(Incong—Shape) × (2-Change − 1-Change) × (2-Change First − 1-Change First)	18.72	14.46	48	1.29	0.2017	0.63	0.47	48	1.34	0.1856

**Table 2:** ANOVA summary of multilevel regressions estimated with maximum likelihood.

	Raw LT (s)					Log-Transformed LT				
*Coefficient Set*	*SS*	*MS*	*Df*	*F*	*p*	*SS*	*MS*	*Df*	*F*	*p*
Familiarization Time	292.07	292.07	(1, 48)	1.40	0.2432	0.24	0.24	(1, 48)	1.10	0.3004
First Test	192.10	192.10	(1, 48)	0.92	0.3428	0.13	0.13	(1, 48)	0.57	0.4534
Condition	1517.13	758.57	(2, 48)	3.63	0.0342	1.54	0.77	(2, 48)	3.51	0.0379
Trial Type	2486.39	2486.39	(1, 48)	11.88	0.0012	2.67	2.67	(1, 48)	12.16	0.0011
First Test × Condition	1172.38	586.19	(2, 48)	2.80	0.0707	1.08	0.54	(2, 48)	2.45	0.0969
First Test × Trial Type	201.50	201.50	(1, 48)	0.96	0.3313	0.20	0.20	(1, 48)	0.92	0.3426
Condition × Trial Type	657.08	328.54	(2, 48)	1.57	0.2185	1.48	0.74	(2, 48)	3.37	0.0427
First Test × Condition × Trial Type	612.55	306.28	(2, 48)	1.46	0.2415	0.82	0.41	(2, 48)	1.88	0.1640

While the model fit to the raw data gives similar results to the classical ANOVA, as expected, the model fit to the log-transformed data gives a different estimate of the Trial Type by Condition interaction; the contribution of the pair of coefficients for the overall interaction was significant (*F*(2, 48) = 3.37, *p* = 0.04). This was driven by a significant change in the size of the difference between looking times to 2-Change and 1-Change trials between the Congruent and Shape-Change conditions, quantified by the Congruent—Shape × 2-Change − 1-Change interaction coefficient (*B* = 0.61, *SE* = 0.23, *t*(48) = 2.59, *p* < 1*e* − 04).

**Fig 5:**
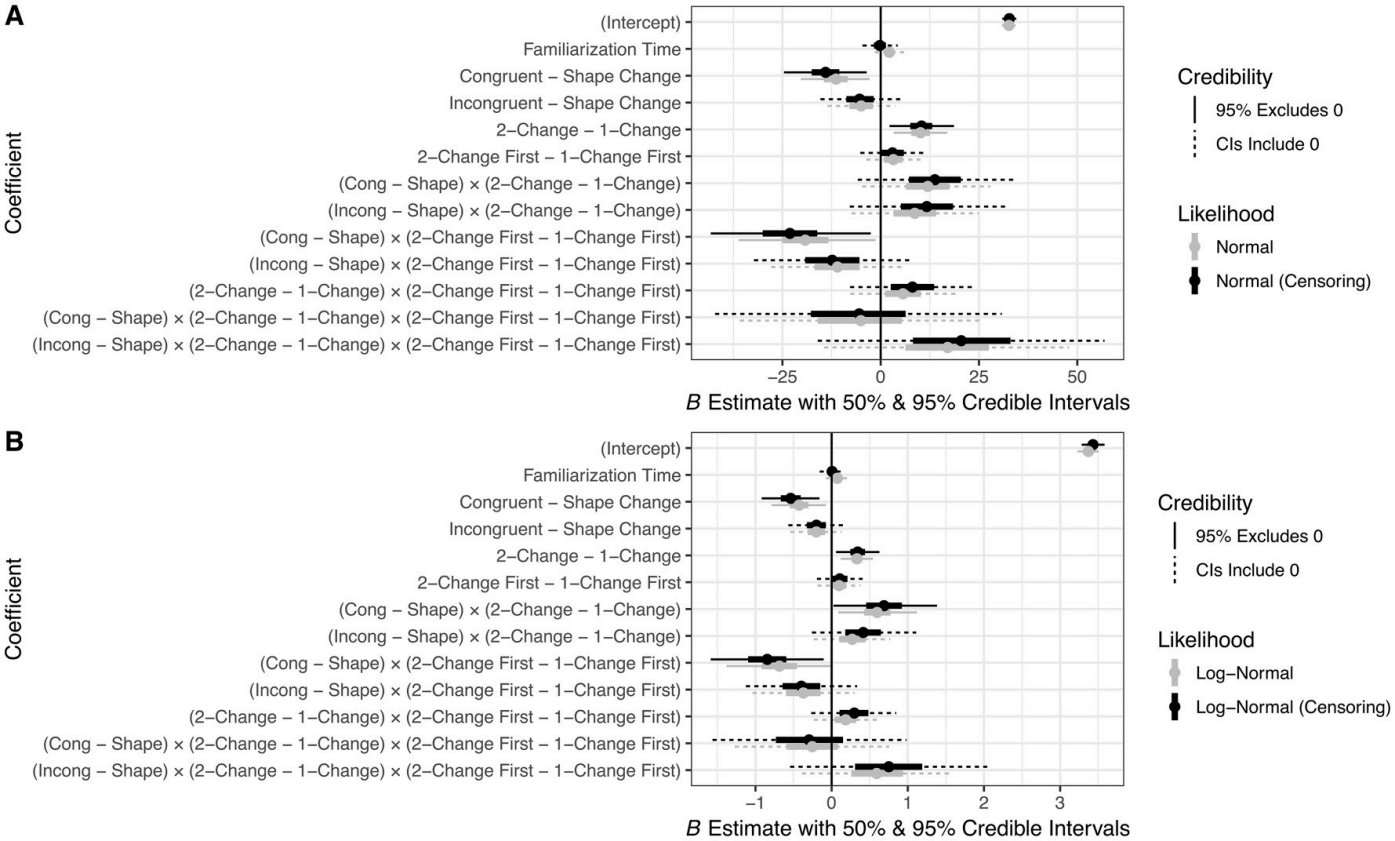
Graphical summary of Bayesian regression coefficients.

**Fig 6:**
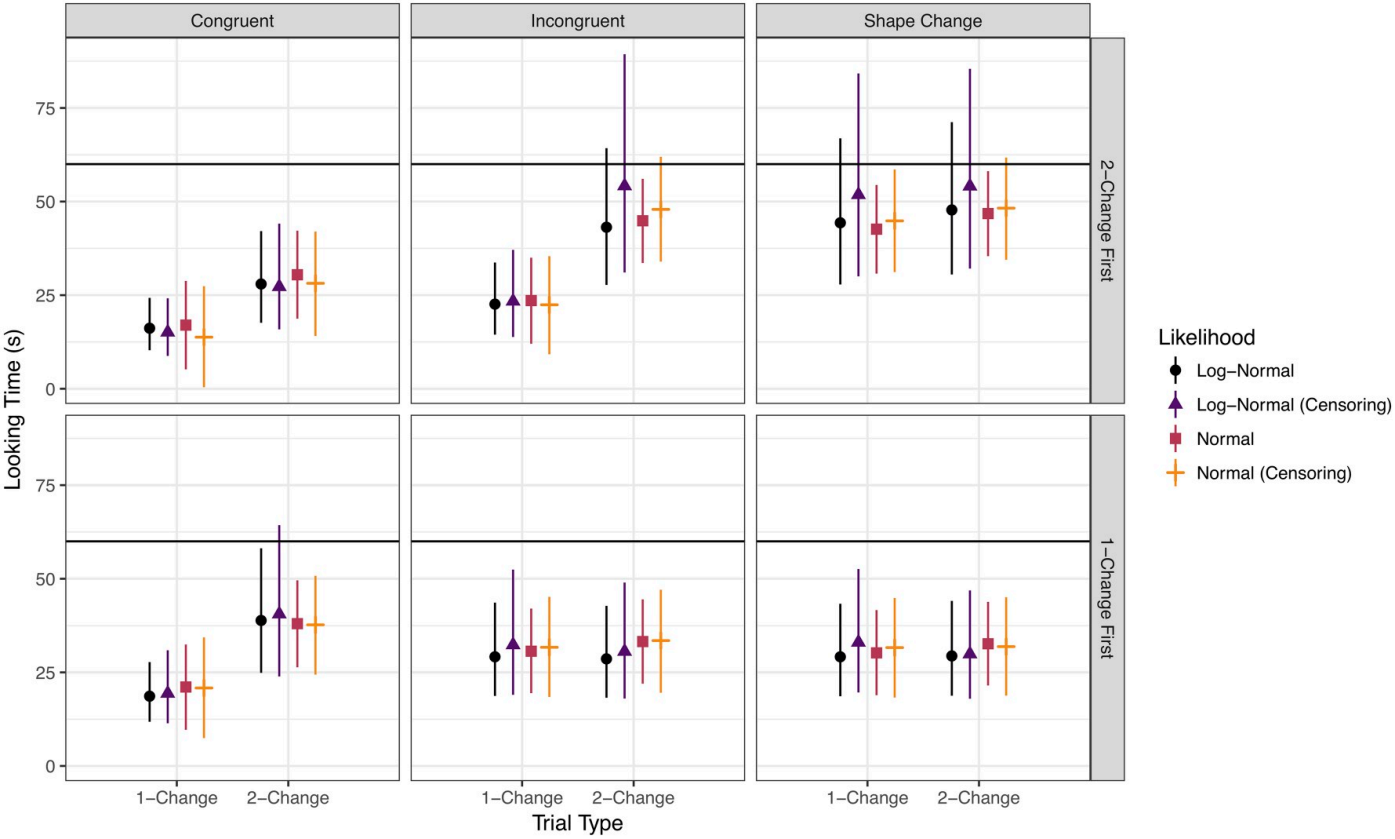
Posterior prediction intervals for looking times under varying likelihood assumptions.

We then examined 4 Bayesian models that varied in likelihood function (normal vs. log-normal) and whether or not the model took censoring into account. In RStan (Stan Development Team, 2018), censoring is treated as a missing data problem: each partially observed (i.e., censored) data point is estimated as an additional parameter whose value is constrained to fall in a given range (here, above 60 *s*) and whose value depends partially on the coefficient parameters estimated from the observable data. Thus, each censored observation has its own posterior distribution. Further details on Bayesian model specification are indicated in Appendices B and C of S1 Appendix. Summaries of the posterior distributions of the coefficients corresponding to each predictor for each of the 4 models are given in Table 3 and graphically summarized in Fig 5. Given that we are using a parameter-estimation approach, we summarize the models with 90% and 95% credible intervals (CIs); a coefficient is said to be credibly different from zero at each of these levels of certainty if these intervals exclude zero. Though there is no direct analogue to the *p*-value in Bayesian hypothesis testing, our approach is similar to hypothesis testing using confidence intervals (for further information on Bayesian intervals, see [36]). In addition, Fig 6 shows the posterior predictive intervals for each model.

**Table 3:** Bayesian model summaries.

	Log-Normal				Normal			
	No Censoring		Censoring		No Censoring		Censoring	
*Coefficient*	*Median*	*95% CI*	*Median*	*95% CI*	*Median*	*95% CI*	*Median*	*95% CI*
(Intercept)	3.37	(3.23, 3.51)	3.43	(3.28, 3.58)	32.60	(30.85, 34.3)	32.72	(30.92, 34.48)
Familiarization Time	0.07	(-0.08, 0.23)	0.01	(-0.16, 0.17)	2.20	(-1.51, 5.95)	-0.20	(-4.62, 4.33)
Congruent—Shape Change	-0.43	(-0.79, -0.08)	-0.54	(-0.92, -0.16)	-11.36	(-20.27, -2.8)	-14.02	(-24.61, -3.55)
Incongruent—Shape Change	-0.20	(-0.54, 0.13)	-0.20	(-0.57, 0.16)	-4.98	(-13.52, 3.69)	-5.36	(-15.33, 5.14)
2-Change − 1-Change	0.33	(0.12, 0.54)	0.34	(0.06, 0.63)	10.14	(3.33, 16.93)	10.40	(2.25, 18.66)
2-Change First − 1-Change First	0.10	(-0.19, 0.38)	0.11	(-0.19, 0.41)	3.25	(-3.66, 10.1)	3.00	(-5.19, 11.08)
(Cong—Shape) × (2-Change − 1-Change)	0.60	(0.09, 1.12)	0.69	(0.02, 1.38)	11.99	(-4.72, 27.91)	13.78	(-5.82, 33.71)
(Incong—Shape) × (2-Change − 1-Change)	0.27	(-0.24, 0.77)	0.42	(-0.26, 1.11)	8.69	(-7.46, 24.93)	11.73	(-7.87, 31.62)
(Cong—Shape) × (2-Change First − 1-Change First)	-0.69	(-1.38, -0.01)	-0.85	(-1.59, -0.1)	-19.20	(-36.16, -1.35)	-23.09	(-43.24, -2.52)
(Incong—Shape) × (2-Change First − 1-Change First)	-0.37	(-1.04, 0.3)	-0.40	(-1.13, 0.33)	-10.99	(-27.85, 5.76)	-12.34	(-32.24, 7.91)
(2-Change − 1-Change) × (2-Change First − 1-Change First)	0.18	(-0.23, 0.6)	0.30	(-0.27, 0.85)	5.68	(-7.72, 19.32)	8.08	(-7.8, 24.13)
(Cong—Shape) × (2-Change − 1-Change) × (2-Change First − 1-Change First)	-0.25	(-1.27, 0.75)	-0.30	(-1.56, 0.98)	-5.05	(-35.81, 25.98)	-5.47	(-42.13, 30.82)
(Incong—Shape) × (2-Change − 1-Change) × (2-Change First − 1-Change First)	0.59	(-0.39, 1.59)	0.75	(-0.55, 2.04)	17.03	(-14.4, 47.89)	20.48	(-16, 56.94)

The models fitted without censoring parallel those fitted with maximum likelihood. In both cases, a Bayesian version of *R*^2^ [37] revealed that the log-normal likelihood provided a better fit to the data (without censoring, normal likelihood: *R*^2^ = 0.33; without censoring, log-normal likelihood: *R*^2^ = 0.48; with censoring, normal likelihood: *R*^2^ = 0.39; with censoring, log-normal likelihood: *R*^2^ = 0.47). Note that these are median values calculated over a full posterior distribution of *R*^2^ values.

**Fig 7:**
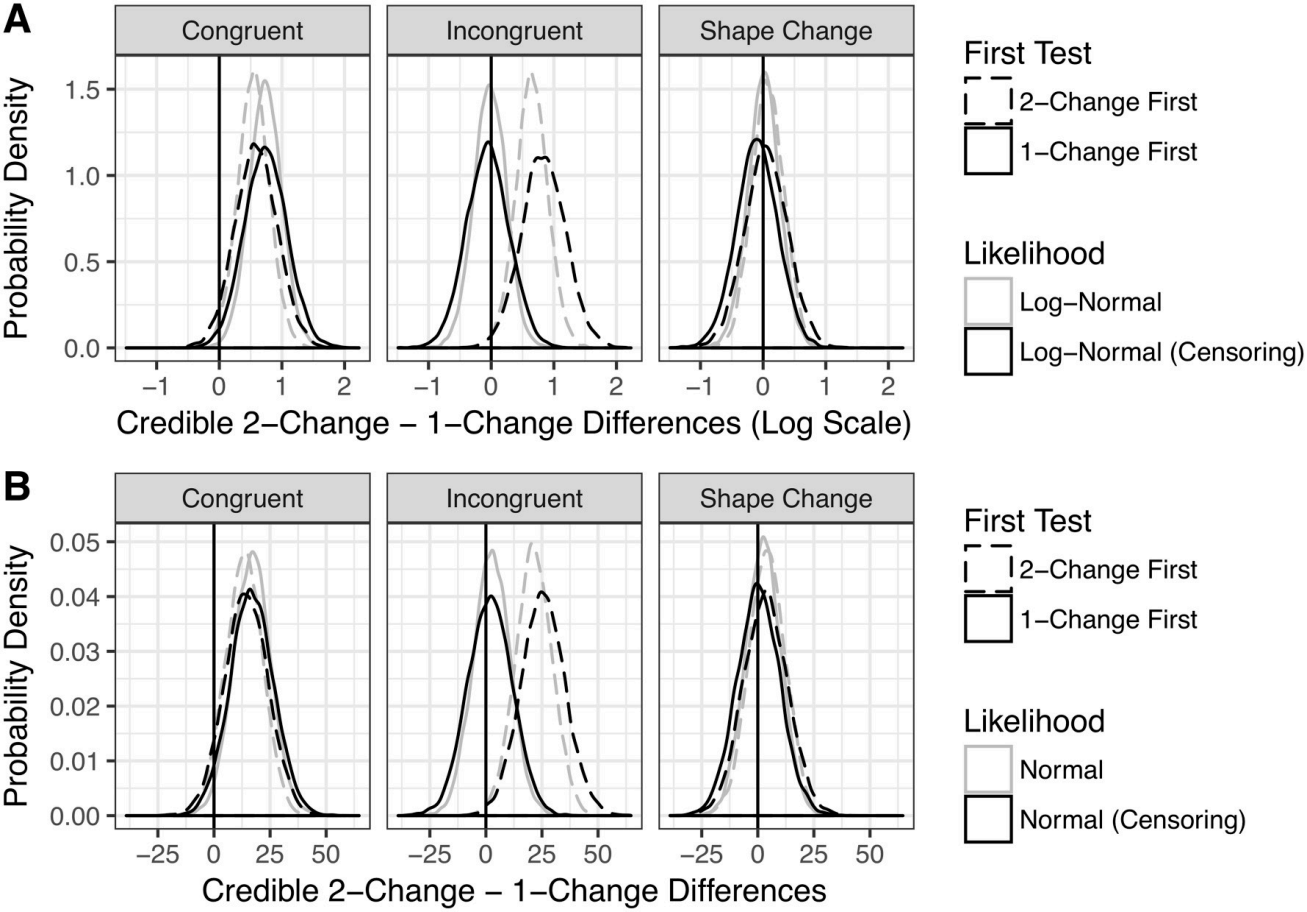
Posterior distribution of within-subject, looking-time differences.

The Bayesian models reaffirmed and strengthened the conclusions drawn from the ANOVA and maximum-likelihood models: newborns credibly preferred 2-Change over 1-Change trials in both trial orders of the Congruent condition and in the 2-Change First order of the Incongruent condition. In contrast, newborns showed no credible difference between 2-Change and 1-Change looking times in either order for Shape Change trials. See Fig 7 for a summary of the posterior distributions of the credible differences in 2-Change and 1-Change trials in each subcondition. In addition, the log-normal models show a credible interaction between the Congruent—Shape Change contrast and the 2-Change − 1-Change contrast, indicating that the difference between 2-Change and 1-Change trials was credibly larger in the Congruent condition than the Shape Change condition, averaging over trial orders.

In general, the models that account for censoring increase the size of the credible intervals around the medians of the coefficients and the predicted subcondition medians. It is important to note that the conclusions we draw withstand this additional safeguard, as the uncertainty intervals around the predictions in the log-normal model increase most dramatically as the median predictions rise.

### Comparison to number-length-duration mappings

A natural follow-up question is whether there are any differences in looking behavior between number/duration-length pairings and number/duration-brightness pairings. Similar behavior across the current experiment and the published experiments would suggest that newborns bring similar expectations to quantities outside size, duration, and number. Dissimilar behavior would suggest that newborns expect specific relations for size, duration, and number that do not extend to brightness (and perhaps other magnitudes).

In the original de Hevia et al. [8] paper, the authors reported that newborn infants preferred 2-Change over 1-Change trials for congruent, but not incongruent, mappings. The authors concluded that newborns mapped number onto length and duration, but did not report any effects of trial order. Here we reanalyze the data from Experiment 1 of de Hevia et al. [8], the most methodologically similar experiment to the current one (see the original paper for methods), in an aggregate model to detect any possible behavioral differences across experiments.

**Fig 8:**
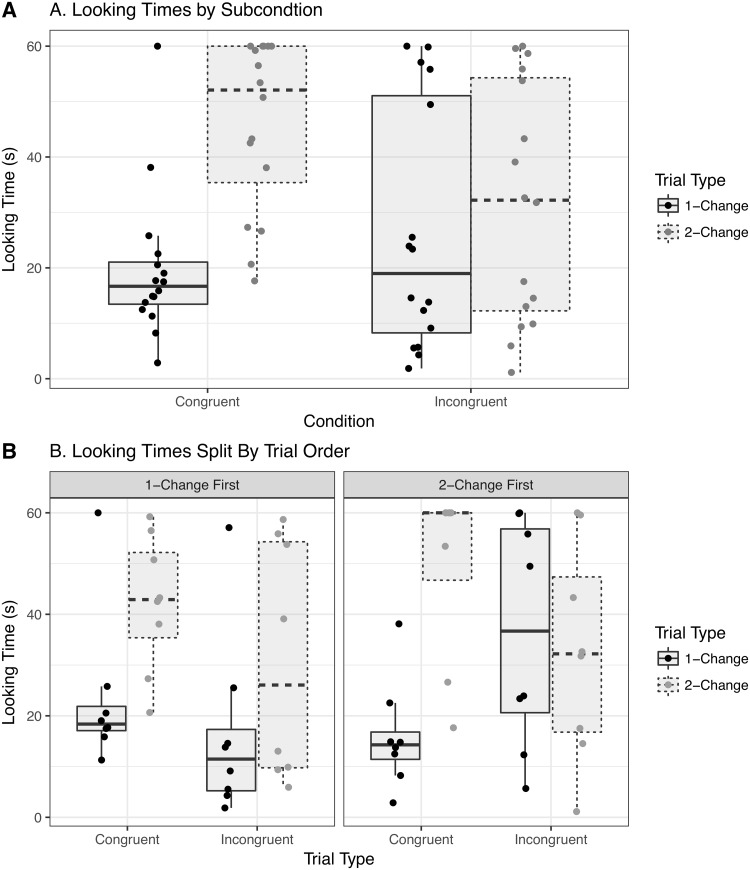
Looking times during test trials of number-length mappings from de Hevia et al. (2014), Experiment 1.

First, we present the raw data from that experiment graphically in Fig 8. Visually, a clear difference between the Congruent and Incongruent conditions can be seen when collapsing across trial order. Infants prefer 2-Change over 1-Change trials only in the Congruent condition. In addition, there does not appear to be any clear effect of trial order in the Incongruent condition. If anything at all, there is a stronger preference for 2-Change trials in the 1-Change First subcondition, which is the *opposite* of the effect seen in the number/duration-brightness mappings, in which a preference for 2-Change trials in the Incongruent condition only occurred in the 2-Change First subcondition.

**Fig 9:**
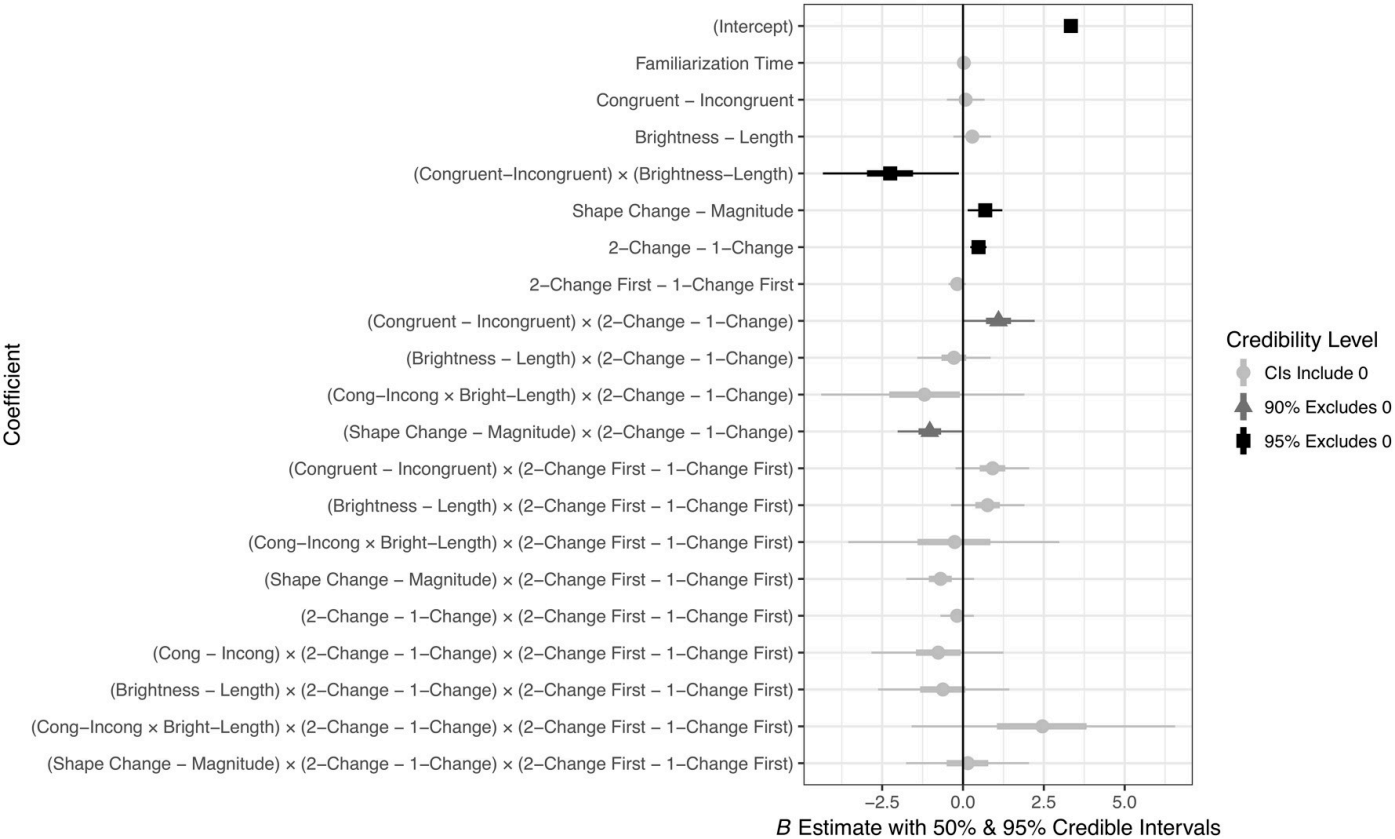
Graphical summary of Bayesian regression coefficients for aggregate model.

**Fig 10:**
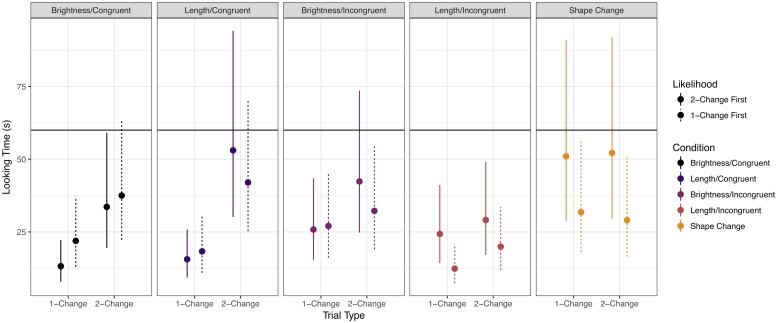
Posterior prediction intervals for looking times for aggregate model.

To investigate any potential differences across experiments, we compiled the data from both experiments and constructed a Bayesian multilevel regression model analogous to those previously discussed, but limited to the censored log-normal likelihood model. The coefficient estimates are displayed in Table 4 and graphically in Fig 9. In addition, the posterior predictive distribution is displayed in Fig 10. Details of model specification are given in Appendices A4 and A5.

**Table 4:** Aggregate model summary.

*Coefficient*	*Median*	*95% CI*
(Intercept)	3.3360451	(3.21, 3.47)
Familiarization Time	0.0271806	(-0.11, 0.17)
Congruent—Incongruent	0.0794864	(-0.5, 0.67)
Brightness—Length	0.2863105	(-0.3, 0.86)
(Congruent-Incongruent) × (Brightness-Length)	-2.2519872	(-4.34, -0.13)
Shape Change—Magnitude	0.6874970	(0.14, 1.22)
2-Change − 1-Change	0.4804811	(0.23, 0.73)
2-Change First − 1-Change First	-0.1797578	(-0.45, 0.09)
(Congruent—Incongruent) × (2-Change − 1-Change)	1.0985407	(-0.01, 2.22)
(Brightness—Length) × (2-Change − 1-Change)	-0.2820748	(-1.41, 0.85)
(Cong-Incong × Bright-Length) × (2-Change − 1-Change)	-1.1926824	(-4.39, 1.9)
(Shape Change—Magnitude) × (2-Change − 1-Change)	-1.0276952	(-2.02, 0)
(Congruent—Incongruent) × (2-Change First − 1-Change First)	0.9148912	(-0.23, 2.05)
(Brightness—Length) × (2-Change First − 1-Change First)	0.7597305	(-0.38, 1.9)
(Cong-Incong × Bright-Length) × (2-Change First − 1-Change First)	-0.2590027	(-3.55, 2.98)
(Shape Change—Magnitude) × (2-Change First − 1-Change First)	-0.6943224	(-1.75, 0.34)
(2-Change − 1-Change) × (2-Change First − 1-Change First)	-0.1838091	(-0.7, 0.34)
(Cong—Incong) × (2-Change − 1-Change) × (2-Change First − 1-Change First)	-0.7659517	(-2.83, 1.25)
(Brightness—Length) × (2-Change − 1-Change) × (2-Change First − 1-Change First)	-0.6228855	(-2.63, 1.43)
(Cong-Incong × Bright-Length) × (2-Change − 1-Change) × (2-Change First − 1-Change First)	2.4559398	(-1.59, 6.56)
(Shape Change—Magnitude) × (2-Change − 1-Change) × (2-Change First − 1-Change First)	0.1466036	(-1.76, 2.04)

Aside from the expected, credible main-effect difference between 2-Change and 1-Change looking times, the (Shape Change—Magnitude) × (2-Change − 1-Change) coefficient’s 95% CI bordered on zero, indicating an overall (marginally) credible difference between 2-Change and 1-Change trials that differed between the magnitude dimensions and the Shape Change control. In addition, the 95% CI for the (Congruent—Incongruent) × (Brightness—Length) coefficient excluded zero, indicating that the looking times to the Brightness stimuli and the Length stimuli differed across their respective Congruent and Incongruent conditions, indicating varying levels of interest to the stimuli across magnitude conditions, averaging across Trial Type. This appears to be driven solely by a difference between looking times in the Incongruent Conditions across dimensions (*M*_*log*_ = 0.43, 95% CI = [0.038, 0.823], averaging over Trial Type and Trial Order).

The (Congruent—Incongruent) × (2-Change − 1-Change) coefficient’s 95% CI also bordered on zero, indicating a marginally stable difference in the size of the difference in looking times to 2-Change and 1-Change trials between Congruent and Incongruent conditions, averaging across magnitude types.

## Discussion

To summarize our findings, newborn infants prefer brightness to change in the same direction as number/duration. However, when brightness and number/duration change in the opposite direction, newborns give a response that depends on trial order: if a 2-Change trial occurs first, newborns prefer 2-Change over 1-Change trials; if a 1-Change trial occurs first, newborns show no such preference.

In contrast, the looking times to the 2-Change trials were similar to looking times to 1-Change trials in the Shape Change control. This difference in looking patterns is unlikely to be explained by higher visual-change salience in brightness than shape conditions for several reasons. First, newborns in the Shape Change condition showed different looking times across trial orders but not across trial types within each order. In particular, looking times to 2-Change-First trials in the shape condition were somewhat higher and less variable than in either of the 2-change brightness conditions. This suggests to us that shape changes may have been somewhat *more* salient than luminance changes to newborns. On the other hand, even if changes were less salient than changes in luminance, and newborns were merely responding additively to the number of feature changes relative to the familiarization trial, we would expect an attenuated—but not eliminated—difference between 2-Change and 1-Change trials. Thus, newborns’ qualitatively different behavior in the Shape Change control indicates that our failure to find a 2-way interaction between the 1-Change vs. 2-Change and Congruent vs. Incongruent manipulations was likely not due to a generic preference to look longer when more than one perceptual feature changes from familiarization.

We interpret this to mean that newborns likely treat simultaneous changes in number/duration and brightness as a potentially related shifts in quantity rather than an unrelated feature change. However, they do not possess a strong preference for these quantities to increase or decrease in parallel. In other words, looking times in the Congruent condition show that newborn expectations are partly similar to those generated in number/duration-space pairings, but looking times in the Incongruent condition suggest a weak, context-dependent divergence from this pattern.

This result appears to contrast with the finding reported in de Hevia et al [8], in which the authors observed a clear difference between behavior in Congruent and Incongruent conditions, with no preference to 2-Change test trials in the latter, indicating a robust dispreference for incongruent number-length pairings. Despite this apparent discrepancy between the Trial Types in the Incongruent conditions of number/duration-length vs. number/duration-brightness mappings, our aggregate reanalysis taking order effects into account failed to confirm this 3-way interaction. However, the 2-way interaction between Brightness vs. Length and Congruent vs. Incongruent manipulations driven by an overall difference in Incongruent looking times suggests that infants behave differently in some way across the dimensions. Nevertheless, the difference in looking times for 2-Change and 1-Change test trials in the Congruent condition was consistent across experiments, indicating that newborns can generate expectations about changes in brightness from changes in number/duration.

Though the strength of the conclusions of our aggregate analysis are limited by our sample size, our results may indicate that the associations among changes in size, duration, and number changes are subtly different from associations between number/duration and brightness changes at birth. Further work is needed to determine the extent to which this generation of expectations differs in strength from expectations generated across the three canonical domains of size, duration, and number, and what contexts (including trial order) contribute to the weaker mappings between brightness and number, length, and duration observed later in development, as well as to the qualitatively different performance with number/duration-length vs. number/duration-brightness mappings at birth described here. In addition, future work will be necessary to determine whether any mappings are handled by a centralized module similar to theoretical constructs like the generalized magnitude system (e.g., [38, 39]) or if apparent specialization for space, time, and number merely reflect emergent similarity among distinct mappings.

One challenge for establishing developmental continuity from birth to adulthood is the implementation of uniform methods; currently it is difficult to draw comparisons across age groups because different methods have been used to elicit behavior signatures in different age groups. The current newborn experiments rely on bimodal stimulation, whereas other experiments with brightness have been presented only visually [22, 23, 25].

A related, theoretical challenge is that different types of reasoning or perceptual systems may underlie the same behavior. There are at least two that may underlie preferences for magnitudes to increase or decrease simultaneously: an analogical system for reasoning about abstract, relative quantities and a system for integrating various sensory signals for estimating latent causal properties of the environment.

Quantities like number, size, duration, loudness, and brightness share abstract descriptions in the form of relative amounts. For instance, a rock can be described as 3 times as large as its neighbor or simply described as larger. Similarly, a light bulb can be described as 3 times as luminous as another or just brighter. In most cases such as these, shared descriptions do not imply shared causal mechanisms: the relative change in light-bulb brightness is completely unrelated to the increase in rock size. Classic, magnitude-estimation tasks show that human adults behave as though they possess such a reasoning system: they can equate proportional changes in magnitude across any dimension or sensory domains given explicit instruction [40]. Moreover, adults can use ratio-based and rank-based representations to judge the similarity of two sequences of stimuli varying in magnitude within and across multiple dimensions [16].

However, some causal mechanisms in the environment *do* result in matching relative magnitudes. For example, an object moving at a constant rate will travel distances proportional to duration; the cumulative surface area of objects will increase with number, given a constant object size. From this perspective, a system for generating expectations across quantitative dimensions would support integrating cues across and within multiple sensory domains to infer the values of latent causes in the environment. If this is the case, then the extent to which expectations are generated from spatial, temporal, and numerical cues to other quantities should be determined by their causal relevance, as has been observed in multisensory integration [41]. For example, number and cumulative surface area are positively correlated in the environment when object size is approximately constant. The relationship between the number of items and their associated brightness depends on context: for example, if the items are light bulbs, then more bulbs would lead to higher brightness levels. However, if the items are dark rocks, then more rocks would lead to lower brightness levels.

### Conclusions

We find that newborn infants prefer an object to change in brightness in parallel with auditory stimuli that change in number and/or duration, but also seem to accept a change in brightness in the opposite direction depending on context. Thus, consistent with previously published experiments with older infants and children, newborns seem to treat brightness as *potentially* related to number/duration, but without a strong expectation for the direction of association. This behavior cannot be explained by a change in the number of changing features, as newborns’ looking behavior differs when shape changes with number/duration. Newborns seem to treat shape differently as if it were an independent source of change, unrelated to number/duration.

Further work is needed to determine under what contexts newborns expect number or duration to change in parallel to brightness, as preferences for changing brightness levels in the opposite direction occurred but were order-dependent. It could be that while newborns have stronger constraints on the kinds of associations that can be formed across number/duration and length than on brightness, they process brightness and shape information independently. Overall, our data provides some evidence for a privileged relationship among length, duration, and number at birth, as relations between number/duration and brightness seem to be somewhat less restricted at birth.

## Supporting information

S1 Appendix Raw data visualization and assumption checks with additional references. (PDF)

S1 Supporting Information Survival analysis of looking times with additional references. (PDF)
