# Supplementary material for: The association of brightness with number/duration in human newborns
Source: PLoS One. 2019 Oct 1;14(10):e0223192. doi: 10.1371/journal.pone.0223192 (PMC6773210; doi:10.1371/journal.pone.0223192)
Supplement: S1 Appendix — (PDF) [file pone.0223192.s001.pdf]

Appendix

All code is available via an OSF repository (Bonn, 2018) at https://osf.io/mskjv/?view_only=9742814098804c10b2622cdaae0d4967.

A Assumption Checks

We first checked the form of the raw distribution of looking times for test trials (Figure 1). The distribution appears to be roughly skewed to the right, consistent with the observation by Csibra et al. (2016) that infant looking times are well described by a log-normal distribution. In addition, a large proportion of observations occurred at 60 s, indicating that when trials ended at that time a number of infants may have been able to continue to maintain gaze at the monitor. In other words, some of the looking times were only partially observed; if this is ignored in statistical analysis, some cell means and their corresponding error variances may be underestimated as well as decreasing the size of any interactions. See the Appendix for additional assumption checks.

In addition to the histogram calculated over raw looking times, Figure 1 shows the best fitting normal and log-normal distributions, calculated with and without accounting for censoring using the fitdistrplus package in R (Delignette-Muller & Dutang, 2015). Without assuming censoring, the AIC value for the best-fitting normal distribution was 835.02 and the AIC value for the best-fitting log-normal distribution was 843.36. Assuming censoring, the AIC value for the best-fitting normal distribution was 767.04 and the AIC value for the best-fitting log-normal distribution was 750.48. The model with the lowest AIC, and thus the best fit, is the censored log-normal. We thus expect that the model that best accounts for the raw data, ignoring any effects of experimental manipulation, will be one fitted with log-normally distributed looking times and that additionally accounts for censoring.

More important for the validity of statistical models than the distribution of the raw looking times is the normality and homogeneity of the *residuals* for interpretation of standard errors (and thus statistical significance tests via F tests). After fitting an analysis of variance of raw looking times as a function of First Test, Trial Type, and Condition, we examined the model residuals as a function of the fitted values to detect any violations of the homogeneity of variances and a quantile-quantile (QQ) plot to assess potential non-normality of the residuals.

The Residuals-vs-Fitted plot in Figure 2A shows an important effect of censoring in which the maximum value of the residuals declines as the fitted values increases. In addition, the lower bound for looking times at 0 causes the minimum residual value to increase as the fitted values decrease. The Q-Q plot in Figure 2B shows deviations from normality at the tails of the distribution that are likely the result of the lower bounds at 0 and 60 s. Taken together, these suggest that adjustments to the classical ANOVA model may be necessary for accurate assessment of uncertainty in the model's estimates.

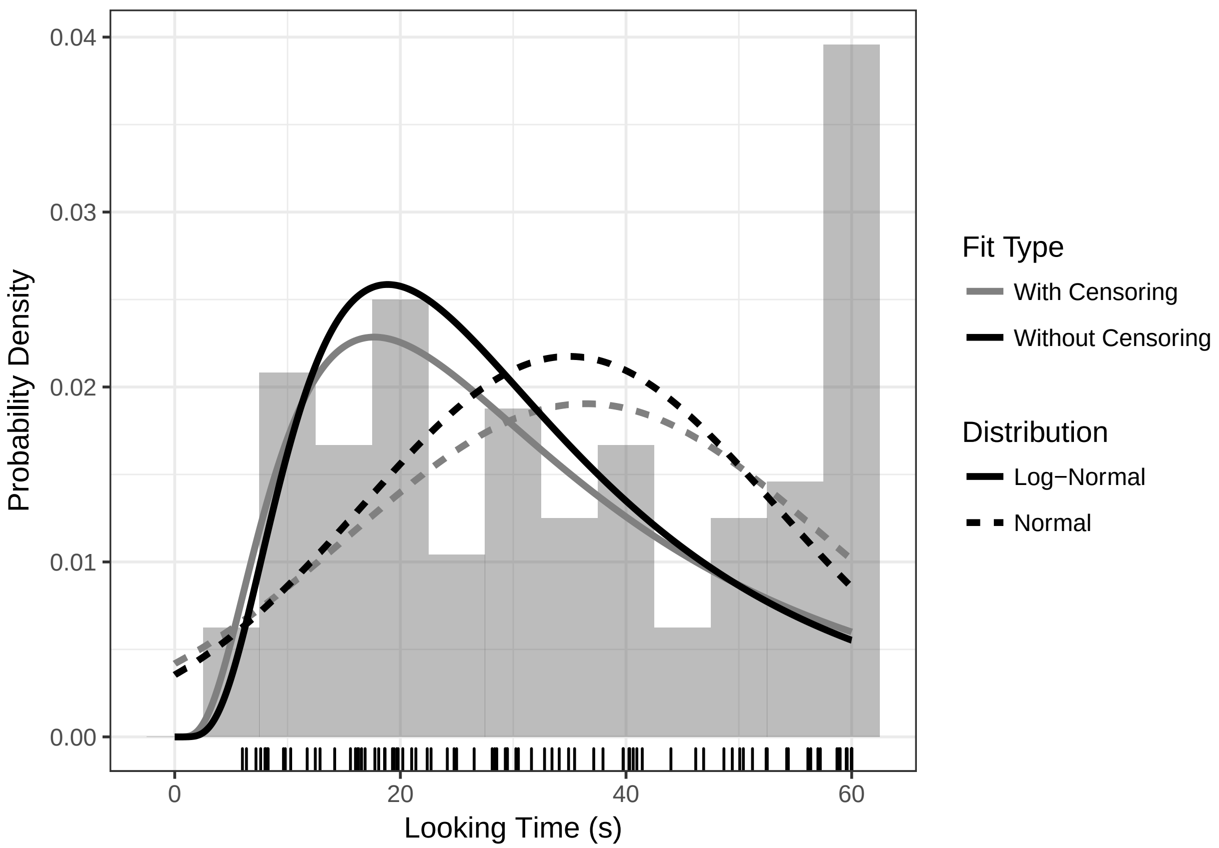

Figure 1: Distribution of looking times. Histogram of raw looking times with best-fitting normal and log-normal distributions superimposed, with and without censoring taken into account. Raw looking times indicated by tick marks beneath the x-axis.

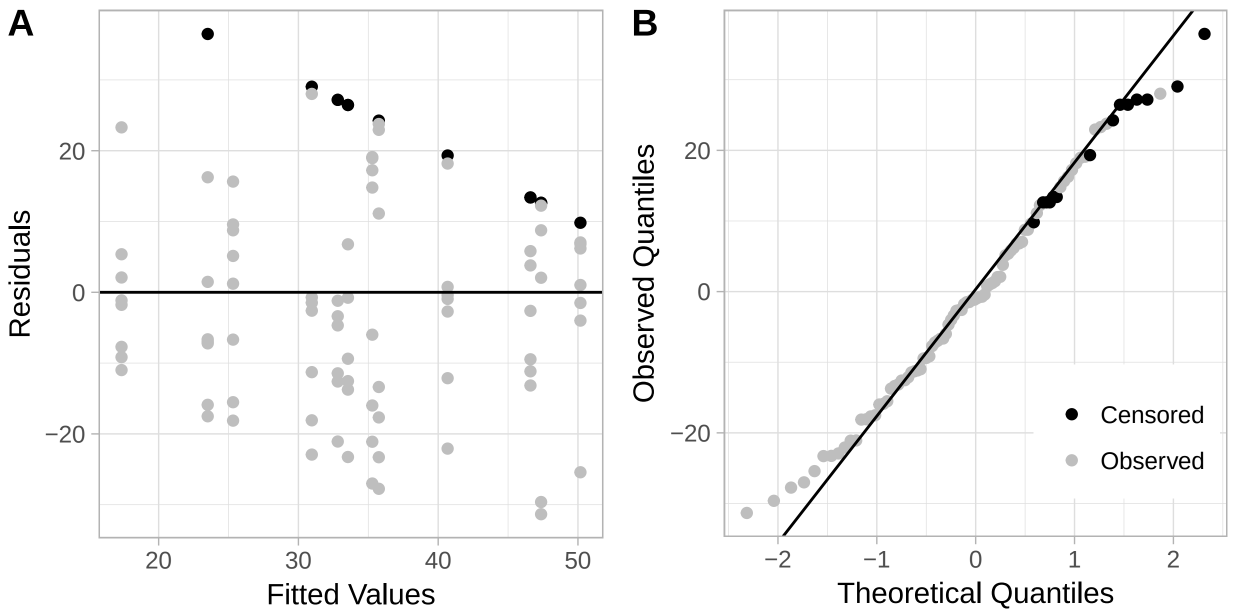

Figure 2: Diagnostics for classical ANOVA model. A. Residuals-vs- fitted plot. B. Quantile-quantile plot.

B Notes on Regression Model for the Current Experiment

We first give an explanation of the basic model structure and how it expands on the classical ANOVA model in the first section. We then give a summary of 6 different models examining the effects of different modifications of approach: Bayesian vs. maximum-likelihood versions of the log-linear and linear models without censoring and Bayesian models with censoring.

Each factor in an ANOVA with number of levels n is coded as $n - 1$ separate predictors in a regression. Each subcondition within each factor corresponds to a unique numerical score given by a standard, centered coding scheme for each predictor. (Centering–subtracting the mean–is essential for the interpretation of the coefficients for main effects in the presence of interactions. Since all predictors were centered, the intercept term corresponds to the grand mean and the meaning of each coefficient corresponds to the effect size of the corresponding predictor, averaging over the others.)

We contrast-coded Trial Type (1-Change = -0.5; 2-Change = 0.5) and First Test (1-Change First = -0.5; 2-Change First = 0.5). Thus, the coefficient for the Trial Type predictor can be interpreted as the average difference in (raw or log) looking times to the two trial types (2-Change - 1-Change). The coefficient for the First Test predictor can be interpreted as the average looking time at test for each of the two types of trial orders (average looking time for 2-Change First newborns - average looking time for 1-Change First newborns).

We simple-coded Condition with the Shape-Change group as the reference group. Simple codes are equivalent to centered dummy codes. Since there were three conditions, this required 2 simple codes; one for which the coefficient reflects the average difference between Congruent and Shape-Change looking times and one for which the coefficient reflects the average difference between Incongruent and Shape-Change looking times. For the first code, rows from the Congruent condition had a score of 2/3 while the other two conditions had a score of $-1/3$. For the second code, rows from the Incongruent condition had a score of 2/3 while the other two conditions had a score of $-1/3$.

Interaction terms for each factor are generated by multiplying each possible pair of codes for each predictor for 2-way interactions and each possible triple of codes for 3-way interactions. For example, the 2-way interaction between Trial Type and Condition is represented by two terms: one representing how the effect of Trial Type may differ between the Congruent and Shape-Change conditions and one representing how the effect of Trial Type may differ between the Incongruent and Shape-Change condition.

We also included z-scored familiarization looking time as a predictor.

In all, the regression model includes 13 coefficients of interest: 1 representing the intercept, 1 representing familiarization time as a covariate, 1 representing Trial Type, 1 representing First Test, 2 representing Condition, 2 representing the interaction between Condition and First Test, 2 representing the interaction between Condition and Trial Type, and 2 3-way interaction terms. In addition, the model included an intercept term for each newborn; these were constrained to be deviations from the main intercept and were modeled as being generated by a normal distribution.

In constructing the regression models, we incorporated the spirit of AN(C)OVA by assigning one prior to each factor. Each batch was assigned a normal prior centered at zero and standard deviation 3 times the residual SD from the data reported in de Hevia et al. (2014), with the following exceptions: (1) since it was not of primary interest, the *Intercept* was assigned a prior with a mean at the empirical mean and a standard deviation of 1, (2) the subjects' individual intercepts were drawn from a normal distribution with mean 0 and the standard deviation was given a Cauchy(0, 1) hyperprior. In addition, to fix issues with divergent transitions in the Hamiltonian Monte Carlo change due to data sparsity, the standard deviation was multiplied by a scaling factor with a standard normal prior (for details on non-centered parameterization, see Betancourt & Girolami, 2013).

C Model for current experiment

LT = Looking Time; β_x = Group-Level Regression Coefficients; τ = Rescaleed Subject-Level Intercept, α = Subject-Level Intercept, σ = Residual Standard Deviation, σ_α = Standard Deviation of Subject-Level Intercepts; i = Data Row Index, j = Subject Index, k = Coefficient Index

$$\begin{aligned}
\hat{LT}_i &= (\beta_0 + \tau_{ji}) + \beta_1 \text{Familiarization}_i + \beta_2 \text{Trial.Type}_i + \beta_3 \text{First.Test}_i + \\
&\quad \beta_{4.1} \text{Congruent.v.Shape}_i + \beta_{4.2} \text{Incongruent.v.Shape}_i + \\
&\quad \beta_{5.1} \text{Trial.Type} \times \text{Congruent.v.Shape}_i + \beta_{5.2} \text{Trial.Type} \times \text{Incongruent.v.Shape}_i + \\
&\quad \beta_{6.1} \text{First.Test} \times \text{Congruent.v.Shape}_i + \beta_{6.2} \text{First.Test} \times \text{Incongruent.v.Shape}_i + \\
&\quad \beta_7 \text{Trial.Type} \times \text{First.Test}_i + \\
&\quad \beta_{8.1} \text{Trial.Type} \times \text{Congruent.v.Shape} \times \text{First.Test}_i + \\
&\quad \beta_{8.2} \text{Trial.Type} \times \text{Incongruent.v.Shape} \times \text{First.Test}_i && (1) \\
\tau_j &= \alpha_j \sigma_\alpha && (2) \\
LT_i &\sim \text{(Log-)Normal}(\hat{LT}_i, \sigma) && (3) \\
\alpha_j &\sim \text{Normal}(0, 1) && (4) \\
\sigma &\sim \text{half-Cauchy}(0, 3SD) && (5) \\
\sigma_\alpha &\sim \text{half-Cauchy}(0, 1) && (6) \\
\beta_k &\sim \text{Normal}(0, 3SD) && (7)
\end{aligned}$$

D Notes on Aggregate Model

To build an analogous regression model for the aggregated data across the current and published work, in addition to contrast-coded Trial Type (1-Change = -0.5 and 2-Change = 0.5), First Test (1-Change First = -0.5 and 2-Change First = 0.5), and z-scored Familiarization Time, we constructed a new coding scheme for Condition to account for the following contrasts across the 5 aggregated groups of 16 infants each with different familiarization types:

1. Congruent ($2 \times 0.25 = 0.5$) vs. Incongruent ($2 \times -0.25 = -0.5$)
2. Brightness ($2 \times 0.25 = 0.5$) vs. Length ($2 \times -0.25 = -0.5$)
3. Congruent vs. Incongruent $\times$ Brightness vs. Length (code 1×2)
4. Magnitude ($4 \times -0.125 = -0.5$) vs. Shape (0.5)

In all, with 2- and 3-way interactions, there were 20 coefficients.

E Aggregate Model

$$
\begin{aligned}
\hat{LT}_i = {} & (\beta_0 + \tau_{ji}) + \beta_1 \text{Familiarization}_i + \beta_2 \text{Trial.Type}_i + \beta_3 \text{First.Test}_i + \\
& \beta_{4.1} \text{Congruent.v.Incongruent}_i + \beta_{4.2} \text{Brightness.v.Length}_i + \\
& \beta_{4.3} \text{Congruent.v.Incongruent} \times \text{Brightness.v.Length}_i + \beta_{4.5} \text{Magnitude.v.Shape}_i + \\
& \beta_{5.1} \text{Trial.Type} \times \text{Congruent.v.Incongruent}_i + \beta_{5.2} \text{Trial.Type} \times \text{Brightness.v.Length}_i + \\
& \beta_{5.3} \text{Trial.Type} \times (\text{Congruent.v.Incongruent} \times \text{Brightness.v.Length})_i \\
& + \beta_{5.4} \text{Trial.Type} \times \text{Magnitude.v.Shape}_i \\
& \beta_{6.1} \text{First.Test} \times \text{Congruent.v.Incongruent}_i + \beta_{6.2} \text{First.Test} \times \text{Brightness.v.Length}_i + \\
& \beta_{6.3} \text{First.Test} \times (\text{Congruent.v.Incongruent} \times \text{Brightness.v.Length})_i + \\
& \beta_{6.3} \text{First.Test} \times \text{Magnitude.v.Shape}_i + \\
& \beta_7 \text{Trial.Type} \times \text{First.Test}_i + \\
& \beta_{8.1} \text{Trial.Type} \times \text{Congruent.v.Incongruent} \times \text{First.Test}_i + \\
& \beta_{8.2} \text{Trial.Type} \times \text{Brightness.v.Length} \times \text{First.Test}_i + \\
& \beta_{8.3} \text{Trial.Type} \times (\text{Congruent.v.Incongruent} \times \text{Brightness.v.Length}) \times \text{First.Test}_i + \\
& \beta_{8.4} \text{Trial.Type} \times \text{Magnitude.v.Shape} \times \text{First.Test}_i
\end{aligned} \tag{8}
$$

$$\tau_j = \alpha_j \sigma_\alpha \tag{9}$$

$$LT_i \sim \text{(Log-)Normal}(\hat{LT}_i, \sigma) \tag{10}$$

$$\alpha_j \sim \text{Normal}(0, 1) \tag{11}$$

$$\sigma \sim \text{half-Cauchy}(0, 3SD) \tag{12}$$

$$\sigma_\alpha \sim \text{half-Cauchy}(0, 1) \tag{13}$$

$$\beta_k \sim \text{Normal}(0, 3SD) \tag{14}$$

F References

Betancourt, M. J., & Girolami, M. (2013). Hamiltonian Monte Carlo for hierarchical models. *arXiv:1312.0906 [Stat]*. Retrieved from http://arxiv.org/abs/1312.0906

Bonn, C. D. (2018). Number-brightness association in human newborns. *Open Science Framework*. Retrieved from https://osf.io/mskjv/?view_only=9742814098804c10b2622cdaae0d4967

Csibra, G., Hernik, M., Mascaro, O., Tatone, D., & Lengyel, M. (2016). Statistical treatment of looking-time data. *Developmental Psychology*, *52*(4), 521–536. https://doi.org/10.1037/dev0000083

de Hevia, M. D., Izard, V., Coubart, A., Spelke, E. S., & Streri, A. (2014). Representations of space, time, and number in neonates. *Proceedings of the National Academy of Sciences*, *111*(13), 4809–4813. https://doi.org/10.1073/pnas.1323628111

Delignette-Muller, M. L., & Dutang, C. (2015). fitdistrplus: An R package for fitting distributions. *Journal of Statistical Software*, *64*(4), 1–34. Retrieved from http://www.jstatsoft.org/v64/i04/
