# Supplementary material for: The association of brightness with number/duration in human newborns
Source: PLoS One. 2019 Oct 1;14(10):e0223192. doi: 10.1371/journal.pone.0223192 (PMC6773210; doi:10.1371/journal.pone.0223192)
Supplement: S1 Supporting Information — (PDF) [file pone.0223192.s002.pdf]

Supporting Information

S1 Survival Analysis of Looking Times

A semi-parametric alternative to traditional, parametric analyses is *survival analysis* (Cox & Oakes, 1984). This analysis does not operate directly on the likelihood, but uses the survival function to estimate group differences in the *instantaneous* probability of trial termination called the *hazard rate*. (A survival function is 1 - the empirical cumulative distribution.) The model does not assume a functional form of the raw distribution of looking times. The classic, proportional hazards model, assumes that group deviations from the baseline hazard rate are multiplicative. We report a survival analysis here that is an analogue of the maximum-likelihood regressions reported in the main text (with the same coding scheme). The subject effects (or frailty effects) are normally distributed, multiplicative deviations from the baseline hazard, analogous to random effects in the maximum likelihood regressions. We used the coxme package to perform the analysis (Therneau, 2018). See Table S1 for the results. The analysis indicates a significant interaction between Trial Type and the Congruent/Shape Change comparison, analogous to the result in the maximum-likelihood model on log-transformed data.

Table S1: Proportional Hazards Model

Coefficient	*B*	*SE*	*z*	*p*
Familiarization Time	-0.1957038	0.1618101	-1.21	0.2300
Congruent/Shape Change	0.6520040	0.3814929	1.71	0.0870
Incongruent/Shape Change	0.0332478	0.3571666	0.09	0.9300
2-Change/1-Change	-0.6756878	0.2454677	-2.75	0.0059
2-Change First/1-Change First	-0.0614909	0.2907328	-0.21	0.8300
(Cong/Shape) × (2-Change/1-Change)	-1.3166640	0.5733159	-2.30	0.0220
(Incong/Shape) × (2-Change/1-Change)	-1.0319339	0.5919495	-1.74	0.0810
(Cong/Shape) × (2-Change First/1-Change First)	1.2251258	0.7241285	1.69	0.0910
(Incong/Shape) × (2-Change First/1-Change First)	0.8532697	0.7075813	1.21	0.2300
(2-Change/1-Change) × (2-Change First/1-Change First)	-0.7419075	0.4785949	-1.55	0.1200
(Cong/Shape) × (2-Change/1-Change) × (2-Change First/1-Change First)	0.0456054	1.1345725	0.04	0.9700
(Incong/Shape) × (2-Change/1-Change) × (2-Change First/1-Change First)	-1.5969892	1.1778397	-1.36	0.1800

S2 References

Cox, D. R., & Oakes, D. (1984). *Analysis of Survival Data. CRC Press.* Retrieved from https://www.crcpress.com/Analysis-of-Survival-Data/Cox-Oakes/p/book/9780412244902

Therneau, T. M. (2018). *Coxme: Mixed effects cox models.* Retrieved from https://CRAN.R-project.org/package=coxme
